# Study of Metal–Semiconductor–Metal CH_3_NH_3_PbBr_3_ Perovskite Photodetectors Prepared by Inverse Temperature Crystallization Method

**DOI:** 10.3390/s20010297

**Published:** 2020-01-05

**Authors:** Lung-Chien Chen, Kuan-Lin Lee, Kun-Yi Lee, Yi-Wen Huang, Ray-Ming Lin

**Affiliations:** 1 Department of Electro-optical Engineering, National Taipei University of Technology, 1, 3 Sec., Chung-Hsiao E. Rd., Taipei 10608, Taiwan; zwl44@yahoo.com.tw (K.-L.L.); wendy930906@gmail.com (Y.-W.H.); 2 Department of Electrical Engineering, China University of Science and Technology, Taipei 11581, Taiwan; kelvin119@gmail.com; 3 Department of Electronics, Chang Gung University, Wen-Hwa 1st Road, Kwei-Shan, Tao-Yuan 33302, Taiwan

**Keywords:** CH_3_NH_3_PbBr_3_ perovskite crystals, inverse temperature crystallization, large-area crystals, MSM photodetectors

## Abstract

Numerous studies have addressed the use of perovskite materials for fabricating a wide range of optoelectronic devices. This study employs the deposition of an electron transport layer of C_60_ and an Ag electrode on CH_3_NH_3_PbBr_3_ perovskite crystals to complete a photodetector structure, which exhibits a metal–semiconductor–metal (MSM) type structure. First, CH_3_NH_3_PbBr_3_ perovskite crystals were grown by inverse temperature crystallization (ITC) in a pre-heated circulator oven. This oven was able to supply uniform heat for facilitating the growth of high-quality and large-area crystals. Second, the different growth temperatures for CH_3_NH_3_PbBr_3_ perovskite crystals were investigated. The electrical, optical, and morphological characteristics of the perovskite crystals were analyzed by X-ray diffraction (XRD), scanning electron microscopy (SEM), ultraviolet-visible spectroscopy, and photoluminescence (PL). Finally, the CH_3_NH_3_PbBr_3_ perovskite crystals were observed to form a contact with the Ag/C_60_ as the photodetector, which revealed a responsivity of 24.5 A/W.

## 1. Introduction

Perovskite is a material that comprises an ABX3 structure. In this molecular formula, A, B, and X represent an alkali metal ion or a methylamine radical (CH_3_NH_3_), a metal cation (Pb_2_, Sn_2_), and a halogen cation (Cl-, Br-, I-), respectively. Compared with organic semiconductor materials, perovskite materials based on organic metal halides exhibit unique optical and electrical properties. It is well-established that the exciton binding energy of perovskite materials is extremely small; therefore, the majority of the excitons, which are generated after being excited by light, can be separated to form free electrons and holes at room temperature. Further, the carrier current possesses a fast diffusion speed and a long diffusion distance. The diffusion lengths of electrons and holes vary with the crystal structure. Compared with MAPbI_3_, MAPbBr_3_ has a shorter lattice constant, higher cohesion energy, lower phase transition temperature, and superior anisotropy [1]. The energy gap is approximately 2.2 eV, and the emission wavelength is green [2]. It has a significantly high optical gain and can be used as a gain dielectric layer in laser [3]. Perovskite materials have been successfully developed in light-emitting diodes [4,5,6,7] and solar cells [8,9,10,11,12]. Perovskite materials can be utilized for a wide range of applications in the field of optoelectronics. Thus far, various types of light sensors have been studied and consequently applied; these include various photodetectors and Schottky barrier diodes [13,14,15,16,17,18,19,20,21,22,23,24,25]. Metal–semiconductor–metal (MSM) structured light sensors or photodetectors have the advantages of a straightforward manufacturing process, high sensitivity, and high response speed as compared with sensors possessing other structures.

Therefore, this study employs CH_3_NH_3_PbBr_3_ perovskite crystals prepared by inverse temperature crystallization method. Conventional crystallization methods, such as the typical cooling or antisolvent vapor-assisted crystallization techniques, are time-consuming and have a long process period. In contrast, the inverse temperature crystallization (ITC) is a fast solution-based crystal growth method, and the optical, electrical, and crystal properties were comparable to the results of them [26,27,28]. C_60_/Ag electrodes were formed to produce a MSM structure for its required research path. The optoelectronic properties of the MSM-type CH_3_NH_3_PbBr_3_ perovskite photodetectors were then examined.

## 2. Materials and Methods

**Figure 1:**
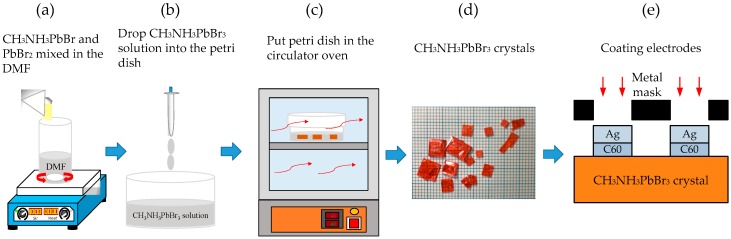
Schematic diagram of experimental procedure in this research.

First of all, the CH_3_NH_3_PbBr_3_ perovskite precursor solution was prepared with the incorporation of 0.0367 g PbBr_2_ (99.998%), 0.0112 g CH_3_NH_3_Br_2_ (MAB, 99.9%), and 1 mL of dimethylformamide (DMF, 98%) solvent. Subsequently, the precursor solution was stirred until it turned clear. The Petri dishes were sonicated with acetone, alcohol, and isopropyl alcohol for 10 min. The precursor solution was then poured into the Petri dish and placed in the hot circulator oven at different temperatures, namely: 40 °C, 50 °C, 60 °C, 70 °C, and 80 °C. CH_3_NH_3_PbBr_3_ crystals were found to grow at a slow rate and gradually became larger during the crystal growth. Finally, the electron transport layer of 20 nm-thick C_60_ and the 100 nm-thick Ag electrodes were deposited on CH_3_NH_3_PbBr_3_ crystals via thermal evaporation and metal mask to complete the MSM structures with an interdigital finger electrode. Figure 1 presents the schematic diagram of the MSM photodetector process procedure.

## 3. Results and Discussion

**Figure 2:**
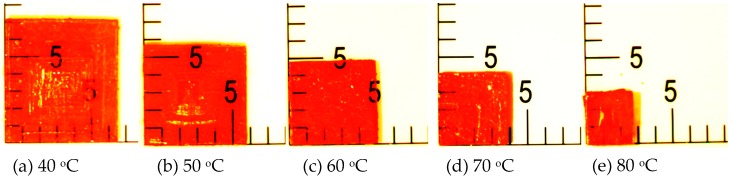
Pictures of MAPbBr_3_ crystals prepared at various temperature. The unit of scale is 1 mm.

In this study, the CH_3_NH_3_PbBr_3_ precursor solution was grown in an oven until the solution completely evaporated. As shown in Figure 2, the CH_3_NH_3_PbBr_3_ crystal size was the largest under the growth temperature at 40 °C (45.5 mm^2^). However, the MAPbBr_3_ crystal area gradually decreased with an increase in the growth temperature. The CH_3_NH_3_PbBr_3_ crystal area was the smallest under the growth temperature at 80 °C (9 mm^2^). Therefore, it can be observed that the growth temperature is inversely proportional to the crystal size. A high temperature makes the solution evaporate and decrease, such that it is difficult to grow a large-sized crystal.

**Figure 3:**
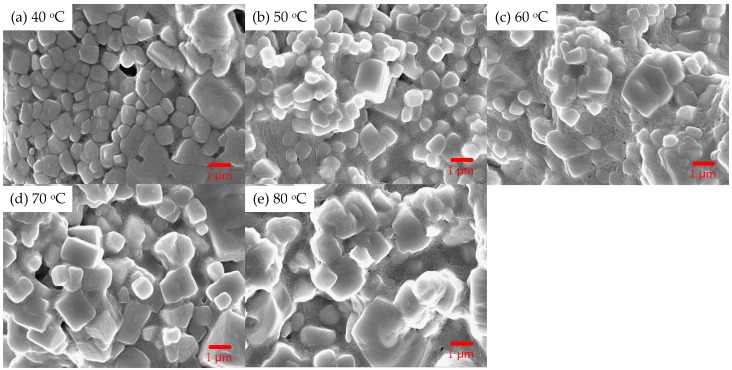
Scanning electron microscopy (SEM) images of the MAPbBr_3_ crystals growth temperature at (**a**) 40 °C, (**b**) 50 °C, (**c**) 60 °C, (**d**) 70 °C, and (**e**) 80 °C.

Figure 3 shows the scanning electron microscopy (SEM) images of the MAPbBr_3_ crystals at 40 °C, 50 °C, 60 °C, 70 °C, and 80 °C, respectively.

The MAPbBr_3_ crystal was composed of several crystal grains. The crystal obtained at 40 and 80 °C comprised many smaller and larger grains, respectively. Further, it was found that the crystal grains constituting the MAPbBr_3_ crystals became larger with an increase in the growth temperature.

**Figure 4:**
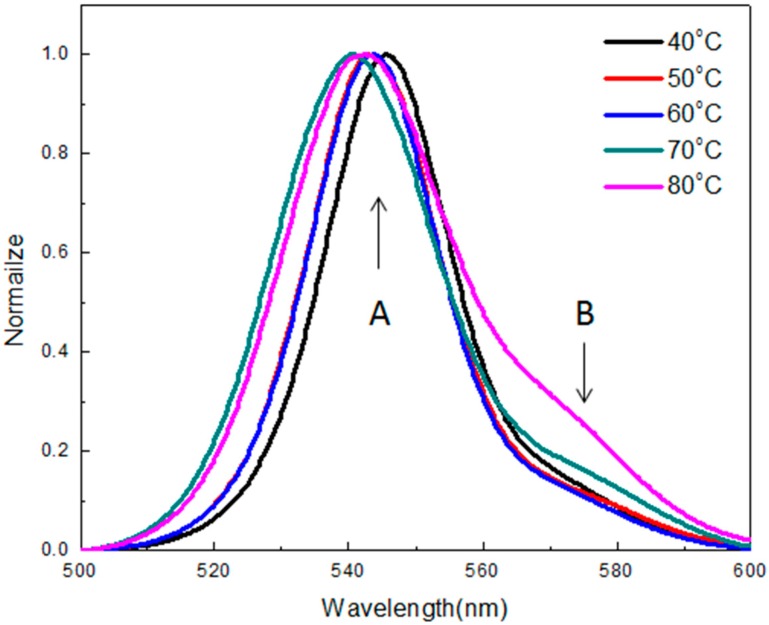
Photoluminescence (PL) spectra of the MAPbBr_3_ crystals prepared at various temperatures.

As shown in Figure 4, the photoluminescence (PL) emission peaks of MAPbBr_3_ crystals were located at 545.6 nm, 543 nm, 543.6 nm, 540.6 nm, and 542.4 nm at temperatures of 40 °C, 50 °C, 60 °C, 70 °C, and 80 °C, respectively. It was observed that the peaks were in close proximity between 540 nm and 546 nm. The PL emission peak exhibited a blue shift with an increase in the growth temperature. The blue shift may be attributed to the difference in laser fluence and measurement system, as well as the atmosphere environment for characterization. [29,30,31]. The dominant PL peak (peak A) with the highest energy is located at ~545 nm (2.275 eV, close to band gap) with a full width at half maximum (FWHM) of ~30 nm. It is corresponding to the band-to-band transition. The lower energy peak (peak B) at ~560 nm, which had a broad bandwidth of 30 nm, was attributed to the emission of band-to-trap state (Br vacancies on the crystal surface) [32,33,34].

**Figure 5:**
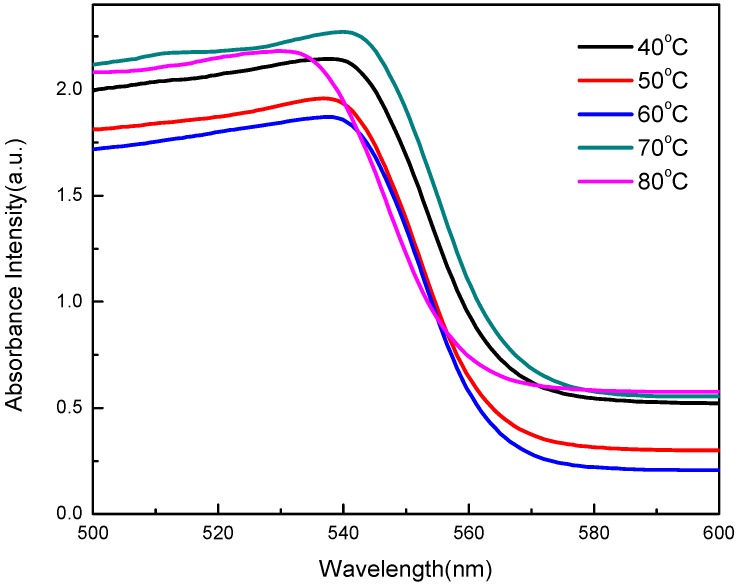
Absorption spectra of the MAPbBr_3_ crystals prepared at various temperatures.

As shown in Figure 5, the edges of absorption spectra of the MAPbBr_3_ crystals were located at 537.58 nm, 536.83 nm, 537.58 nm, 539.85 nm, and 536.03 nm at temperatures of 40 °C, 50 °C, 60 °C, 70 °C, and 80 °C, respectively. It can be observed that the edges of absorption spectra are extremely close to one another, and that the peaks are located between 530 nm and 540 nm, corresponding to the band gap of 2.275 eV of MAPbBr_3_ single crystals [35].

**Figure 6:**
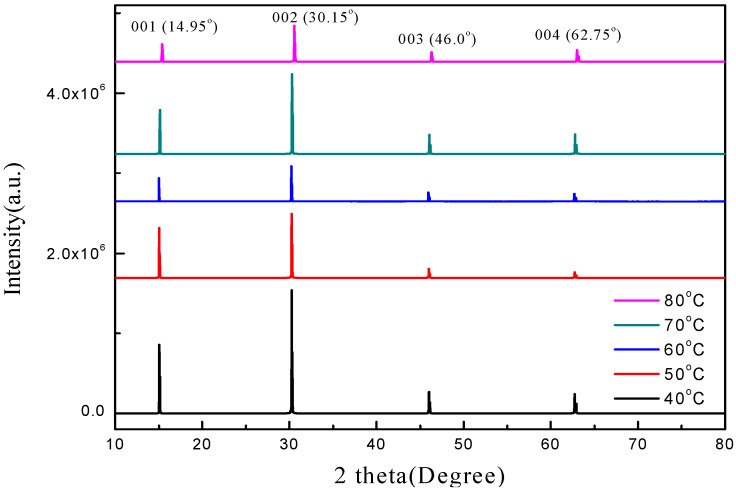
X-ray diffraction (XRD) patterns of the MAPbBr_3_ crystals prepared at various temperatures.

Figure 6 shows the X-ray diffraction (XRD) patterns of the MAPbBr_3_ crystal at 40 °C, 50 °C, 60 °C, 70 °C, and 80 °C. All growth temperatures displayed significant peaks at 14.95°, 30.15°, 46.0°, and 62.75°. The crystal plane directions corresponding to the cubic crystal structure were (001), (002), (003), and (004), which, in turn, correspond to high-quality MAPbBr_3_ crystals. When the temperature was 40 °C, the peak intensity was observed to be higher. Conversely, at 80 °C, the peak intensities were significantly lower. It can be seen from the SEM images in Figure 2 that the crystallinity and density of the sample prepared at 40 °C is the best. The peak intensity is greater than that of the samples prepared at the temperatures of 50 to 80 °C, which is due to the lower density even though the particles are larger.

**Figure 7:**
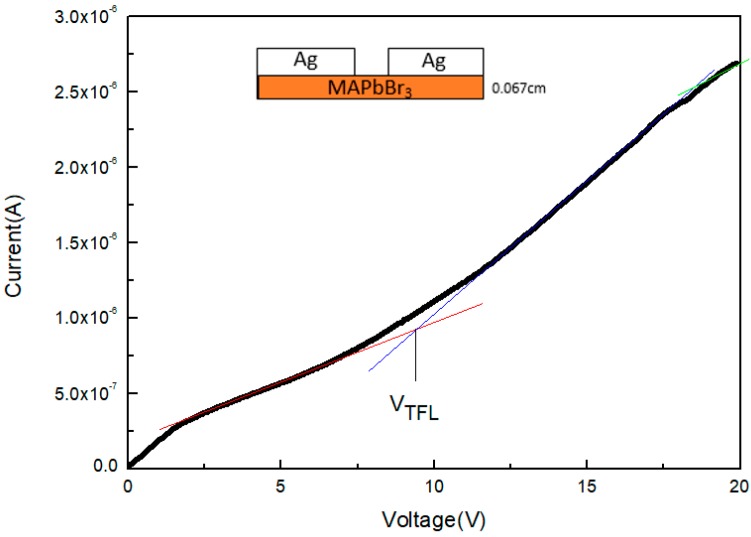
Current–voltage curve of the MAPbBr_3_ crystal.

Figure 7 shows the current–voltage curve of the MAPbBr_3_ crystal; the red, blue, and green lines depict the Ohmic region (*n* = 1), trap-filled region (*n* > 3), and Child’s region (*n* = 2), respectively. According to Mott-Gurney’s law: μ = 8J_D_L^3^/9εε_o_V^2^ [26,36]. Consequently, the carrier mobility of the MAPbBr_3_ crystal was calculated to be 14.4 cm^2^V^−1^s^−1^. The trap density is calculated using the following equation: n_t_ = 2V_TFL_εε_o_/eL^2^; the trap density of MAPbBr_3_ crystals was 4.7 × 10^10^ cm^−3^.

**Figure 8:**
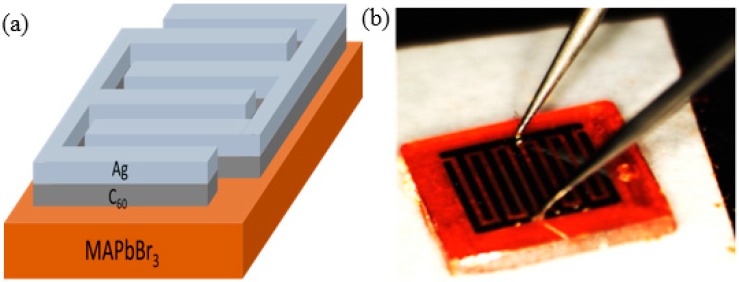
(**a**) Metal–semiconductor–metal (MSM) structure of the MAPbBr3 crystal photodetector. (**b**) Photograph of a MAPbBr_3_ crystal photodetector.

The MSM structure of the MAPbBr_3_ crystal photodetector is shown in Figure 8a. A medium layer C_60_ was inserted between the silver (Ag) electrodes and the MAPbBr_3_ crystal in order to prevent from a compound of both. Figure 8b shows a photograph of the MAPbBr_3_ crystal with the MSM structure.

**Figure 9:**
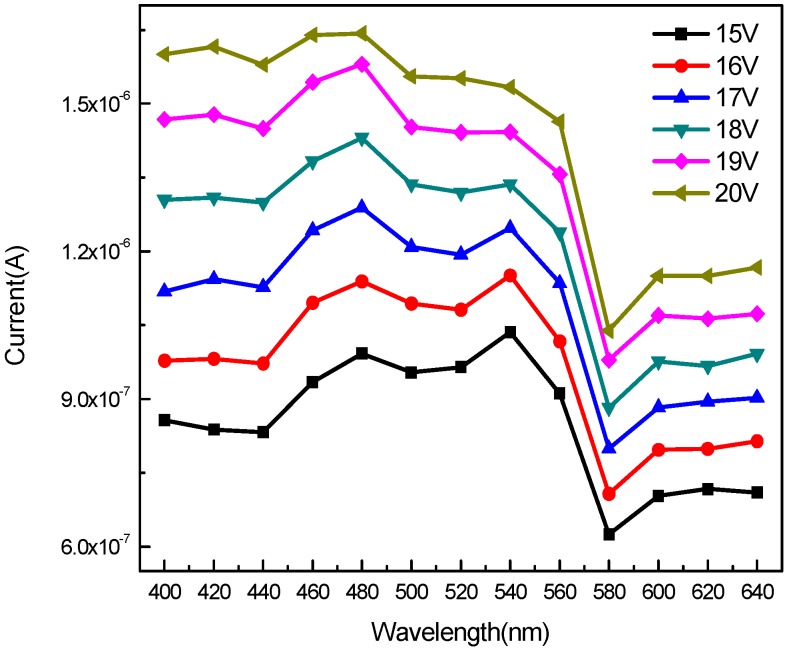
Current and wavelength curve MSM-MAPbBr_3_ crystal photodetectors.

Figure 9 shows the current vs. wavelength graph of the photodetector under each bias. The devices exhibit high current values in the wavelength range of 400 nm to 560 nm for each bias. However, the current decreased significantly in the range of 570 nm to 580 nm. The absorption edge located at around 580 nm is corresponding to the trap level transition. The current was observed to rise slightly between 600 nm and 640 nm owing to the carrier relations from the trap in the band structure.

**Figure 10:**
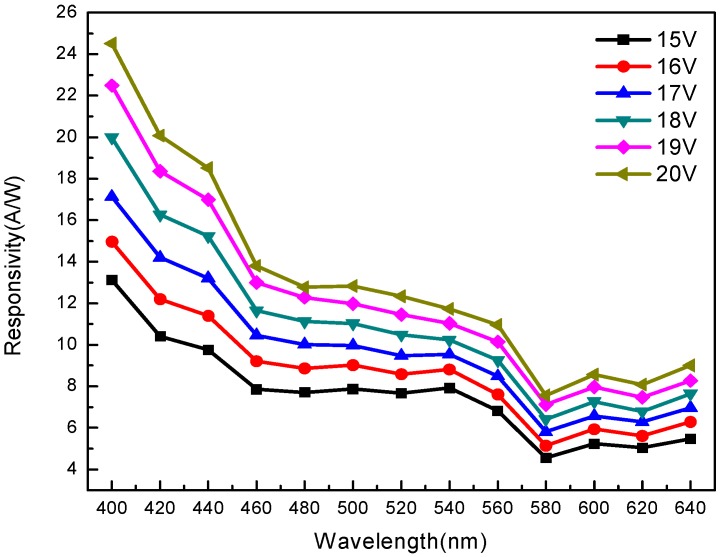
Responsivity and wavelength curve of MSM-MAPbBr_3_ crystal photodetectors.

Figure 10 shows the wavelength and responsivity at different bias voltages. At a wavelength of 400 nm, the responsivity of the component is 13.13 A/W, 14.97 A/W, 17.13 A/W, 19.98 A/W, 22.48 A/W, and 24.50 A/W at different bias voltages of 15 V, 16 V, 17 V, 18 V, 19 V, and 20 V, respectively. From 400 nm to 460 nm, the responsivity gradually decreases and becomes stable in the range of 460 nm to 560 nm. The responsivity attained its lowest value at 580 nm, and increases slightly in the range of 600 nm to 640 nm.

**Figure 11:**
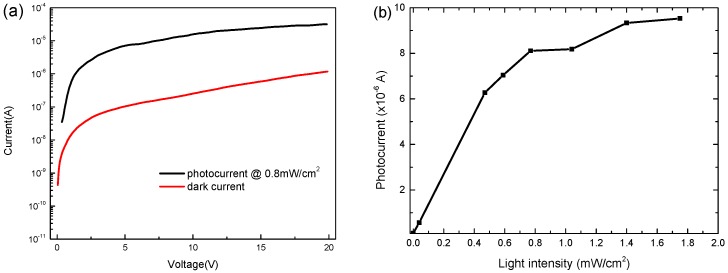
(**a**) Current–Voltage (I–V) characteristics and (**b**) dynamic range of the MSM-MAPbBr3 crystal photodetector under illumination of 0.8 mW/cm^2^ and measured at 5 V.

Figure 11a plots typical dark and illuminated (under 0.8 mW/cm^2^) I–V characteristics of MSM-MAPbBr_3_ crystal photodetectors at a bias ranging from 0 to 20 V. The photocurrent was approximately 7.04 × 10^−6^ A, and the dark current was approximately 1.04 × 10^−7^ A at a bias of 5 V. In this research, a relatively high dark current is a result of leakage current from the boundary in the crystal. However, a large photocurrent-to-dark-current contrast ratio is observed—at almost two orders of magnitude. The orders of magnitude of the photocurrent-to-dark-current contrast ratio were similar to other structures in other studies [37,38,39]. As shown in Figure 11b, the photocurrent density at −5 V was measured under different illumination intensities of a 200 W Xe lamp as the light source in order to study the dependence of the photocurrent on the incident light intensity. A linear relationship was observed when the intensity of incident light power was lower than 0.8 mW/cm^2^. However, when the light intensity was higher than 0.8 mW/cm^2^, the photocurrent was saturated owing to the balance between generation and recombination of electron–hole pairs.

## 4. Conclusions

In this study, MAPbBr_3_ crystals were grown at low temperatures using an improved inverse temperature crystallization method. It was observed that the small single crystals obtained at different growth temperatures became larger as the temperature increased; however, the crystal size was found to decrease as the temperature increased. In all single crystals, the sample prepared at temperature 80 °C showed the smallest size; the single crystal prepared at 40 °C was observed to have the largest size. The XRD pattern revealed four significantly high peaks, which were associated with high-quality MAPbBr_3_ crystals. The PL emission peak was obtained between 536 nm to 538 nm. The absorption edge was located at 580 nm and corresponded to the trap-level transition. The photocurrent increased slightly from 600 to 640 nm, which had been caused by the carrier relations from the trap level.
